# Enhanced recovery after surgery nursing pathway and prognosis assessment in lung cancer patients: A retrospective clinical study

**DOI:** 10.1097/MD.0000000000047899

**Published:** 2026-03-20

**Authors:** Yanmei Chen, Dequn Chen, Yayuan Wang, Yuanqiang Zhang

**Affiliations:** a Department of Cardiothoracic Surgery, Zigong First People’s Hospital, Zigong City, Sichuan Province, China.

**Keywords:** enhanced recovery after surgery, length of stay, lung cancer, perioperative care, postoperative complications, propensity score matching

## Abstract

The value of applying enhanced recovery after surgery (ERAS) pathways in the perioperative management of lung cancer requires further high-level evidence. This study aimed to evaluate the impact of the ERAS nursing pathway on the recovery and prognosis of patients undergoing radical surgery for lung cancer. A retrospective cohort study design was adopted, including patients who underwent radical lung cancer surgery between January 2022 and January 2024. Based on the nursing model, patients were divided into an ERAS group (n = 121) and a conventional care control group (n = 170). Propensity score matching was used to control for confounding factors, resulting in 104 well-matched patients (52 in each group) for analysis. The ERAS group received multidisciplinary, standardized perioperative interventions. Hospital stay, recovery indicators, complications, quality of life (QoL), and patient experience were compared between the 2 groups. After matching, the postoperative hospital stay and total hospital stay in the ERAS group were significantly shorter than those in the control group (median: 5.0 days vs 8.0 days, *P* < .001; 9.0 days vs 13.0 days, *P* < .001). The ERAS group showed significantly earlier times to first ambulation, flatus, oral intake, and chest tube removal (all *P* < .001). Furthermore, the ERAS group had significantly lower overall complication rates (15.4% vs 36.5%, *P* = .012) and pulmonary complication rates (9.6% vs 25.0%, *P* = .035). Additionally, the ERAS group exhibited significantly lower postoperative pain scores, lower incidence of nausea and vomiting, while patient satisfaction and early postoperative QoL scores were significantly higher (all *P* < .05). Subgroup and sensitivity analyses yielded consistent results, confirming the robustness of the conclusions. For lung cancer patients undergoing radical surgery, implementing the ERAS nursing pathway can safely and effectively accelerate postoperative recovery, significantly shorten hospital stay, reduce the risk of complications, and improve patients’ symptom experience and QoL, demonstrating significant clinical value for widespread promotion.

## 1. Introduction

Lung cancer is one of the malignancies with the highest incidence and mortality rates worldwide, and its disease burden is increasingly severe.^[1]^ According to the latest cancer statistics, the annual number of new cases and deaths from lung cancer consistently rank among the highest for all cancers, posing a significant challenge to public health systems.^[2]^ In this context, radical surgical resection remains the cornerstone of lung cancer treatment and is the most critical approach for achieving long-term survival and potential cure in patients with early-stage and carefully selected cases of locally advanced disease.^[3]^

However, traditional perioperative management strategies have demonstrated considerable limitations in long-term clinical practice.^[4]^ These conventional approaches are often associated with a range of significant physiological and psychological stress responses, including surgical trauma stress, adverse effects of anesthetics, inadequate postoperative pain control, metabolic disturbances from prolonged fasting, and unnecessary immobilization.^[5]^ These factors interact, creating a vicious cycle that ultimately leads to a markedly increased risk of postoperative complications, prolonged hospital stays, and delayed functional recovery.^[6]^ This situation not only exacerbates patients’ physical and psychological distress and healthcare costs but may also delay subsequent necessary adjuvant therapies, thereby negatively impacting long-term treatment outcomes and quality of life (QoL) on multiple levels.^[7]^

In response to these clinical challenges, the enhanced recovery after surgery (ERAS) concept was developed. Enhanced recovery after surgery constitutes a multimodal, evidence-based clinical pathway that integrates various optimized perioperative measures.^[8]^ Its core principle is to minimize the stress response induced by surgical trauma and maintain physiological homeostasis throughout the perioperative period, thereby accelerating postoperative recovery.^[9]^ Currently, the ERAS concept has been widely adopted in various surgical specialties, such as colorectal surgery and hip and knee arthroplasty. Substantial high-quality evidence confirms its effectiveness in reducing hospital length of stay, lowering postoperative complication rates, and improving patient satisfaction.^[10]^

In recent years, with advancements in minimally invasive surgical techniques and refined anesthesia management, the application of ERAS pathways in thoracic surgery has progressively gained traction. Preliminary clinical observations suggest that implementing ERAS principles in patients undergoing pulmonary resection may yield positive clinical benefits.^[8]^ Nonetheless, the current body of evidence has notable limitations. There is a lack of systematic evaluation regarding the comprehensive benefits of ERAS for lung cancer surgery patients, particularly its effects on short-term recovery indicators, long-term QoL, and survival prognosis.^[9]^ Most available clinical studies are small-scale observational studies or single-center experiences. Furthermore, due to the inherent limitations of retrospective study designs, baseline characteristics often differ significantly between patient groups receiving different care models. These methodological weaknesses substantially compromise the reliability of the findings and their value for broader clinical implementation.^[10]^

Based on this research background, this study employs a retrospective cohort design and innovatively utilizes propensity score matching (PSM), an advanced statistical method, to effectively control for confounding biases arising from imbalanced baseline data. This approach aims to provide a more scientific and accurate assessment of the impact of a standardized ERAS nursing pathway compared to conventional care in lung cancer patients undergoing radical surgery. The evaluation comprehensively covers multiple dimensions, including hospital stay, postoperative recovery indicators, complication incidence, patient-reported experiences, and short-term QoL. We anticipate that this methodologically rigorous study will provide higher-level clinical evidence to support the standardized application and promotion of ERAS in the perioperative management of lung cancer, ultimately contributing to enhanced recovery quality for patients undergoing lung cancer surgery.

## 2. Materials and methods

### 2.1. Study design

This study was approved by the Ethics Committee of Zigong First People’s Hospital. This study adopted a retrospective cohort design, collecting clinical data from lung cancer patients diagnosed and treated in our hospital from January 2022 to January 2024. The requirement for written informed consent was waived due to the retrospective nature of the study and the use of de-identified data. To minimize the confounding effects of different surgical methods and underlying diseases, and to maintain relative simplicity and homogeneity of study variables, only patients undergoing radical surgery for lung cancer were included, totaling 291 cases. According to whether patients received enhanced recovery after surgery (ERAS) pathway management during the perioperative period, they were divided into 2 groups: enhanced recovery after surgery nursing group (ERAS group): patients who received standardized ERAS nursing measures throughout the entire perioperative period, 121 cases; control group: patients who received traditional conventional care models, 170 cases.

**Figure 1.**
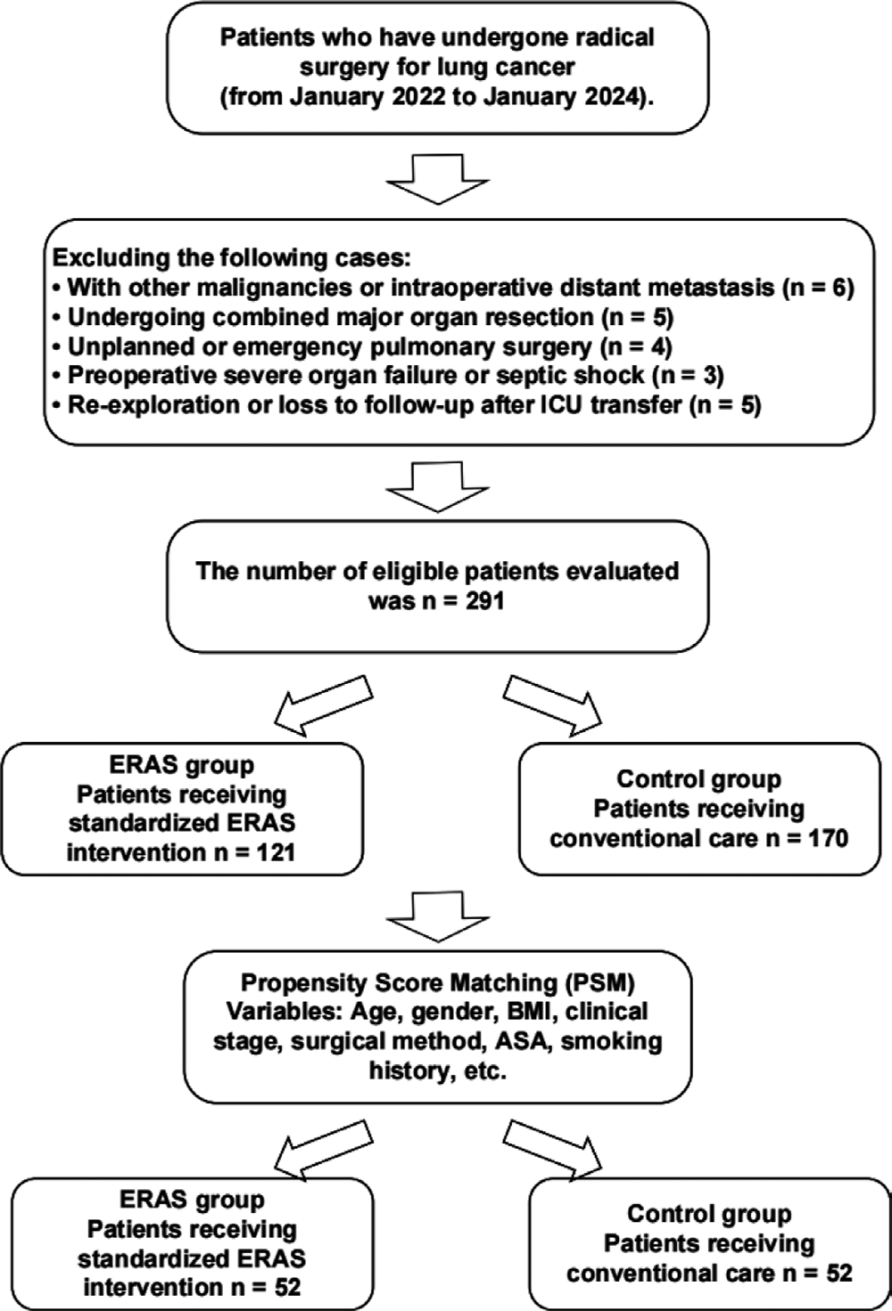
Patient screening and inclusion process. ASA = American Society of Anesthesiologists, BMI = body mass index, ERAS = enhanced recovery after surgery, ICU = intensive care unit, PSM = propensity score matching.

To control for potential confounding in baseline characteristics between the 2 groups, this study used PSM for balance processing. Using general demographic characteristics, surgical type, comorbidity status, etc, as covariates, a propensity score (PS) model was established. Following the 1:1 nearest neighbor matching principle with a caliper value set at 0.2, matching was performed. Finally, 52 successfully matched patients from each group, totaling 104 cases, were obtained for subsequent analysis. The overall patient screening process is shown in Figure 1.

### 2.2. Inclusion and exclusion criteria

Inclusion criteria: Based on the study objectives and characteristics of retrospective cohort research, patients meeting the following conditions were initially screened for inclusion: Age ≥ 18 years, regardless of gender; preoperative definitive diagnosis of primary lung cancer confirmed by pathology or imaging; underwent radical lobectomy, segmentectomy, or sleeve resection at our hospital; preoperative assessment indicating surgical tolerance (ASA grade I–III); possession of complete clinical medical records, surgical records, and nursing information; underwent at least 90 days of postoperative follow-up with complete data.

Exclusion criteria: From the pool meeting the above inclusion criteria, patients with the following conditions were further excluded: Presence of malignant tumors in other sites or intraoperative discovery of distant metastasis; concurrent surgery on other major organs (such as combined heart, esophageal, or mediastinal resection); underwent unplanned pulmonary surgery due to emergency, trauma, or infectious diseases; preexisting severe heart, liver, or renal failure or septic shock; required re-thoracotomy postoperatively or were lost to follow-up after transfer to intensive care; death during hospitalization or loss to follow-up within 90 days postoperatively; incomplete clinical or nursing records preventing acquisition of key variables; not enrolled in or failing to strictly implement the standardized ERAS pathway, with unclear nursing records.

### 2.3. Nursing methods

Based on whether patients received the enhanced recovery after surgery (ERAS) nursing pathway during the perioperative period, the study subjects were divided into the ERAS group and the control group.

The ERAS group was managed by a multidisciplinary team consisting of thoracic surgeons, anesthesiologists, specialized nurses, nutritionists, and rehabilitation therapists. Standardized, evidence-based nursing interventions were implemented across the preoperative, intraoperative, and postoperative stages. The nursing goals focused on reducing postoperative stress response and promoting early functional recovery, covering key aspects such as nutritional optimization, respiratory function exercise, pain control, early mobilization, and complication prevention and control.

The control group received conventional nursing procedures, following traditional thoracic surgical preoperative preparation and postoperative recovery management, primarily characterized by passive recovery, and lacking a systematic rehabilitation pathway and quantitative standards. A detailed comparison of the nursing measures between the 2 groups is shown in Table 1.

**Table 1:** Comparison of nursing measures.

Nursing phase	Nursing measures for the enhanced recovery after surgery (ERAS) group	Nursing measures for the control group
Preoperative care	① Health education: Beyond routine admission education, the responsible nurse provides systematic education on the ERAS concept, explaining the goals, implementation process, and expected benefits. Distributes the “ERAS Patient Education Manual” and “Postoperative Rehabilitation and Pain Management Guide” to enhance patient initiative and participation	① Conducts routine admission education, focusing primarily on intraoperative cooperation and basic precautions
	② Comprehensive assessment: In addition to routine admission assessments, performs nutritional screening (NRS 2002 score), and venous thrombosis risk assessment (Caprini score). Provides oral or enteral nutritional support for patients with a nutritional score < 3, and uses elastic stockings or intermittent pneumatic compression pumps for those with a thrombosis risk score ≥1	② Performs only routine admission assessments, without nutritional or thrombosis risk screening
	③ Preoperative functional preparation: Instructs and supervises patients in pulmonary function exercises (balloon blowing, pursed-lip breathing, diaphragmatic breathing for 10–20 min/session, 3–5 times/d) and encourages walking training to improve lung expansion capacity	③ Follows doctor’s orders for preoperative fasting (12 h for solids, 8 h for liquids), without providing preoperative carbohydrate drinks or nutritional support
	④ Preoperative fasting and fluid management optimization: Fasting for 6 h and clear liquids for 4 h preoperatively, with 200 mL of carbohydrate drink given 2 h before surgery to reduce insulin resistance	④ No systematic pulmonary function exercises preoperatively; patients are only advised to perform deep breathing exercises postoperatively
	⑤ Preoperative psychological intervention: Nurses communicate with patients to alleviate anxiety, correct misconceptions about postoperative pain, and build confidence	⑤ No targeted psychological counseling, only general preoperative communication
	⑥ Preoperative preparation: Performs skin cleaning in the surgical area 1 d before surgery, without mechanical bowel preparation	⑥ Routine skin preparation in the surgical area
	⑦ Completes nursing assessments and documentation	⑦ Completes routine nursing records
Postoperative care	① Monitoring and observation: Closely monitors vital signs, incision, and drainage postoperatively, maintaining ECG monitoring and tube patency.	① Routine monitoring of vital signs and drainage tube care
	② Multidisciplinary rounds: Conducts daily joint rounds involving doctors, nurses, and anesthesiologists to dynamically adjust analgesia, nutrition, and rehabilitation plans	② Only separate physician rounds; nursing follow-up is not systematic
	③ Early mobilization: Guides patients in ankle pump exercises (5 min/session, 5 times/d) after regaining consciousness from anesthesia. Assists patients to sit up or move to the bedside within 6 h, ambulate for 1–2 h on postoperative day 1, 4–6 h on day 2, and perform independent activities from day 3 onwards	③ Guides bedside activities within 24 h of anesthesia recovery, without an individualized rehabilitation plan
	④ Fluid management: Administers individualized intravenous fluids, dynamically adjusted based on urine output, oral intake, and laboratory values to promote fluid balance	④ Administers intravenous fluids according to standard orders, without dynamic adjustment
	⑤ Early oral intake: Allows small amounts of water intake after regaining consciousness. If no nausea or abdominal distension, initiates liquid diet on day 1, transitioning to a regular diet by days 2–3	⑤ Fasting for 24 h postoperatively, starting oral intake only after bowel sounds return
	⑥ Pain management: Implements standardized pain assessment (NRS scale) and adopts a multimodal analgesia strategy (regional block + oral NSAIDs + opioids if necessary). Immediate intervention is provided for pain scores >4	⑥ Pain management primarily based on doctor-ordered medication, lacking systematic assessment
	⑦ Respiratory and pulmonary care: Encourages patients to perform breathing exercises and effective coughing every 2 h, using an incentive spirometer to prevent atelectasis	⑦ Low frequency of respiratory training, with poor patient compliance
	⑧ Complication prevention and control: Nurses focus on monitoring abnormal signs like fever, persistent air leak, and dyspnea for early intervention	⑧ Complication observation relies mainly on patient complaints, lacking quantitative records
	⑨ Recording and follow-up: Strictly records daily recovery indicators using the ERAS bedside surgical nursing form. Provides individualized health education and home rehabilitation guidance before discharge	⑨ Routine nursing records and discharge education

ECG = electrocardiogram, ERAS = enhanced recovery after surgery, NRS = Numeric Rating Scale, NSAIDs = non-steroidal anti-inflammatory drugs.

### 2.4. Data collection

This study collected clinical data from patients meeting the inclusion and exclusion criteria by retrospectively reviewing the hospital’s electronic medical record system, surgical anesthesia system, and nursing records. All data were independently extracted and cross-checked by 2 uniformly trained researchers to ensure accuracy and completeness. Discrepancies were resolved through discussion or adjudication by a third researcher.

#### 2.4.1. Baseline data collection

To assess the comparability between the 2 patient groups and provide covariates for PSM, we systematically collected patient baseline data. Specifically, this included: demographic characteristics such as age, gender, and body mass index (BMI); lifestyle habits such as smoking history (defined as cumulative smoking of more than 100 cigarettes or regular smoking for more than 6 months); disease-related characteristics, including tumor pathological type (adenocarcinoma, squamous cell carcinoma, etc) determined from postoperative pathology reports and preoperative clinical tumor, node, metastasis staging according to the International Association for the Study of Lung Cancer 8th edition standards; surgery-related characteristics, covering surgical approach (lobectomy, segmentectomy, sleeve resection), side of surgery, duration of surgery (time from skin incision to suture completion, in minutes), and intraoperative blood loss (in milliliters); as well as physical status, including American Society of Anesthesiologists (ASA) classification and comorbidities (such as hypertension, diabetes, and chronic obstructive pulmonary disease).

#### 2.4.2. Perioperative outcome measures collection

To comprehensively evaluate the short-term effects of the enhanced recovery nursing pathway, we collected indicators reflecting recovery efficiency and safety. The primary indicators were postoperative hospital stay (postoperative hospital stay as the number of days from the date of surgery to discharge) and total hospital stay (total hospital stay as the number of days from hospital admission to discharge). Recovery efficiency indicators included: time to first ambulation (hours from surgery end to first standing or walking), time to first flatus (hours from surgery end to first anal exhaust), time to first oral intake (hours from surgery end to first oral liquid intake), chest tube duration (days), and total postoperative hospitalization costs (RMB). Safety indicators focused on recording all complications occurring from post-surgery until 30 days after discharge, with diagnoses based on clinical, imaging, and laboratory evidence, and severity assessed using the Clavien–Dindo classification system; specific complications included pulmonary complications (pneumonia, atelectasis, respiratory failure, persistent air leak > 5 days), cardiovascular complications (arrhythmia, heart failure), and other complications (surgical site infection, deep vein thrombosis/pulmonary embolism, anastomotic leakage, etc), while reoperation rate and 30-day readmission rate were also recorded. Time to first ambulation was obtained from postoperative nursing records documenting the first supervised standing or walking event; time to first flatus and first oral intake were extracted from daily nursing progress notes and physician orders recorded in the electronic medical record system.

#### 2.4.3. Patient experience and symptom control data collection

To assess patients’ subjective feelings and symptom burden, we collected relevant experience and symptom indicators. Postoperative pain intensity was assessed using the Numeric Rating Scale (0–10 points), recording scores at rest and during activity at 24, 48, and 72 hours postoperatively. Simultaneously, the incidence of postoperative nausea and/or vomiting (PONV) within 72 hours was recorded. On the day of discharge, patients were invited to rate their overall nursing experience during this hospitalization using a 0 to 10 point scale (0 representing very dissatisfied, 10 representing very satisfied).

#### 2.4.4. Long-term prognosis and quality of life data collection

To investigate the impact of the enhanced recovery pathway on patients’ long-term outcomes, we conducted a 90-day postoperative follow-up. Patients’ survival status was confirmed through outpatient review or telephone contact, and the 90-day postoperative survival rate was recorded. Additionally, the Chinese versions of the European Organisation for Research and Treatment of Cancer Quality of Life Core Questionnaire (EORTC QLQ-C30) and its lung cancer-specific module (QLQ-LC13) were used to assess patients’ QoL at 3 time points: upon discharge, 30 days postoperatively, and 90 days postoperatively. This study focused on the global health status/QoL and physical functioning scales from the QLQ-C30, as well as lung cancer-specific symptom domains such as cough and dyspnea from the QLQ-LC13.

#### 2.4.5. Data preparation for subgroup and sensitivity analyses

To ensure analytical depth and the robustness of the results, we prespecified variables for subgroup and sensitivity analyses. Subgroup analysis variables included age (divided into 2 groups using 65 years as the cutoff), primary surgical method (lobectomy vs segmentectomy), and tumor stage (stage I vs stages II–III). Data for sensitivity analysis involved retaining the complete baseline dataset and primary outcome measures (postoperative hospital stay) for all 291 patients prior to PSM, to enable verification using a multivariate regression model.

### 2.5. Propensity score and sample size validity analysis

#### 2.5.1. Propensity score matching

To control for confounding bias, this study employed PSM. Using whether patients received ERAS management as the dependent variable, baseline variables such as age, gender, BMI, clinical stage, and surgical method were included in a logistic regression model to calculate the PS for each patient. Subsequently, a 1:1 nearest neighbor matching method (caliper value = 0.2) was used to match control group patients to ERAS group patients. After matching, the standardized mean differences for all baseline variables were <0.1, and the *P*-values for intergroup comparisons were all >.05 (see Table 2), indicating good matching effectiveness and achieved balance between the groups.

**Table 2:** Comparison of patient baseline characteristics.

Baseline characteristic	Before matching		Before matching, *P*-value	After matching		After matching, *P*-value
	ERAS group (n = 121)	Control group (n = 170)		ERAS group (n = 52)	Control group (n = 52)	
Propensity score	0.42 ± 0.18	0.58 ± 0.21	<.001	0.48 ± 0.16	0.49 ± 0.15	.735
Demographics						
Age (yr)	61.2 ± 8.5	65.1 ± 9.0	<.001	63.5 ± 8.2	64.0 ± 8.8	.745
Gender (male)	65 (53.7%)	105 (61.8%)	.165	28 (53.8%)	30 (57.7%)	.693
BMI (kg/m^2^)	23.8 ± 2.5	22.8 ± 3.1	.003	23.2 ± 2.6	23.1 ± 2.8	.874
Smoking history (yes)	40 (33.1%)	80 (47.1%)	.015	20 (38.5%)	22 (42.3%)	.693
Disease-related						
Pathological type			.328			.856
Adenocarcinoma	89 (73.6%)	115 (67.6%)		38 (73.1%)	36 (69.2%)	
Squamous cell carcinoma	25 (20.7%)	45 (26.5%)		11 (21.2%)	13 (25.0%)	
Other	7 (5.8%)	10 (5.9%)		3 (5.8%)	3 (5.8%)	
Clinical stage			.007			.864
Stage I	85 (70.2%)	90 (52.9%)		30 (57.7%)	28 (53.8%)	
Stage II	30 (24.8%)	60 (35.3%)		16 (30.8%)	18 (34.6%)	
Stage III	6 (5.0%)	20 (11.8%)		6 (11.5%)	6 (11.5%)	
Surgery-related						
Surgical approach			.018			.901
Lobectomy	110 (90.9%)	140 (82.4%)		44 (84.6%)	43 (82.7%)	
Segmentectomy	8 (6.6%)	25 (14.7%)		6 (11.5%)	7 (13.5%)	
Sleeve resection	3 (2.5%)	5 (2.9%)		2 (3.8%)	2 (3.8%)	
Surgery side (left)	55 (45.5%)	85 (50.0%)	.441	25 (48.1%)	24 (46.2%)	.843
Operation duration (min)	145.0 (120.0–175.0)	150.0 (125.0–180.0)	.455	147.5 (122.5–172.5)	148.0 (125.0–175.0)	.912
Intraoperative blood loss (mL)	100 (80–150)	110 (85–160)	.301	105 (80–150)	100 (80–155)	.785
Physical status						
ASA classification			.002			.816
Grade I–II	105 (86.8%)	122 (71.8%)		40 (76.9%)	38 (73.1%)	
Grade III	16 (13.2%)	48 (28.2%)		12 (23.1%)	14 (26.9%)	
Comorbidities						
Hypertension	38 (31.4%)	65 (38.2%)	.221	17 (32.7%)	18 (34.6%)	.832
Diabetes mellitus	15 (12.4%)	28 (16.5%)	.32	7 (13.5%)	8 (15.4%)	.785
COPD	12 (9.9%)	25 (14.7%)	.217	6 (11.5%)	5 (9.6%)	.752

ASA = American Society of Anesthesiologists, BMI = body mass index, COPD = chronic obstructive pulmonary disease, ERAS = enhanced recovery after surgery.

#### 2.5.2. Sample size validity analysis

The final analytical sample size after PSM in this study was 104 cases (52 cases each in the ERAS group and the control group). To evaluate the statistical power of this sample size for detecting intergroup differences in the primary outcome measure (postoperative hospital stay), a retrospective power analysis was conducted. Using the median postoperative hospital stay of 5.0 days in the ERAS group and 8.0 days in the control group after matching, along with their distribution characteristics as parameters, the power was calculated using the Mann–Whitney *U* test. With α = 0.05 (2-sided), the analysis results showed that the statistical power (1 − β) for detecting this difference under the current sample size was >90%, far exceeding the traditional threshold of 80%. This indicates that the post-matching sample size is sufficient to identify significant differences in the primary outcome between the 2 groups, and the study conclusions are supported by reliable statistical validity.

### 2.6. Statistical analysis

This study used SPSS 26.0 (IBM [International Business Machines Corporation], Armonk) and R 4.2.1 software (The R Core Team; R Foundation for Statistical Computing, Vienna, Austria, https://www.r-project.org) for statistical analysis. All tests were 2-sided, and a *P* < .05 was considered statistically significant. The analysis was performed on the matched data (n = 104). Continuous variables conforming to a normal distribution were described as mean ± standard deviation, and intergroup comparisons were made using independent samples *t*-tests. Those not conforming to a normal distribution were described as median (interquartile range), and intergroup comparisons were made using the Mann–Whitney *U* test. Categorical variables were described as counts (percentages), and intergroup comparisons were made using the chi-square test or Fisher exact test. For repeated measures data such as pain scores and QoL scores, repeated measures analysis of variance was used to assess the main effects of time, the main effects of group, and the interaction effects between time and group. If a significant interaction effect was present, independent samples *t*-tests were further used for intergroup comparisons at each time point. To assess the risk of complications, the relative risk (RR) and its 95% confidence interval (CI) were calculated. To examine the consistency of the ERAS effect across different populations, subgroup analysis was performed with postoperative hospital stay as the outcome, and interaction tests were used to evaluate effect differences. To verify the robustness of the main results, a multivariate linear regression analysis was conducted using the full pre-matching sample (n = 291), and the effect of ERAS was reevaluated after adjusting for confounding factors.

## 3. Results

### 3.1. Balance of patient baseline data

**Figure 2.**
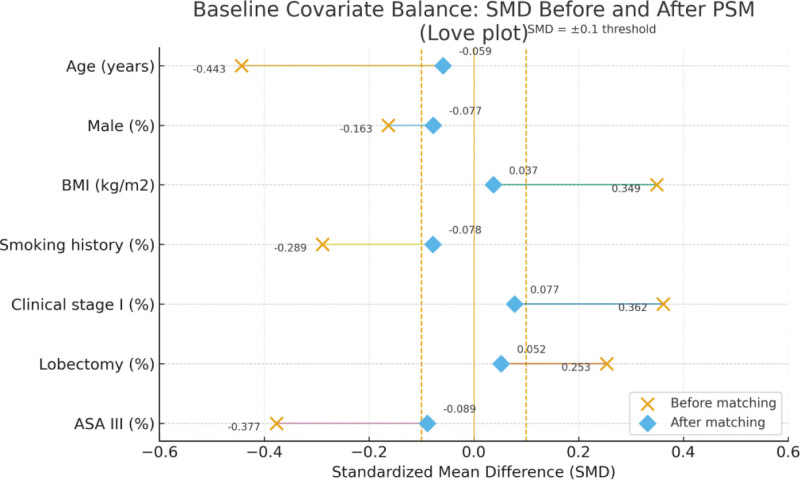
Standardized mean differences (SMDs) of baseline covariates before and after propensity score matching (Love plot). The plot shows covariate balance between the ERAS and control groups before (cross) and after (diamonds) 1:1 nearest-neighbor matching (caliper = 0.2). Dashed lines indicate the ±0.1 threshold for acceptable balance. Most variables exceeded this threshold before matching but achieved good balance after matching, with all SMDs <0.1. ASA = American Society of Anesthesiologists, BMI = body mass index, ERAS = enhanced recovery after surgery, PSM = propensity score matching, SMD = standardized mean differences.

The comparison of baseline data after matching is shown in Table 2 and Figure 2. Before matching, there were statistically significant differences (all *P* < .05) between the 2 groups in several baseline characteristics, including the PS, age, BMI, smoking history, clinical stage, surgical approach, and ASA classification, indicating the presence of significant selection bias. After 1:1 nearest neighbor matching, the differences in all baseline characteristics, including the PS itself, were no longer statistically significant (all *P* > .05) between the 2 groups, confirming the effectiveness of the matching and establishing the comparability of the 2 patient groups.

### 3.2. Comparison of perioperative recovery efficiency

The ERAS group demonstrated comprehensive superiority over the control group in perioperative recovery efficiency (Table 3). Regarding the primary indicators, both the postoperative hospital stay and total hospital stay were significantly shorter in the ERAS group compared to the control group (median: 5.0 days vs 8.0 days, *P* < .001; 9.0 days vs 13.0 days, *P* < .001). In terms of recovery efficiency indicators, the time to first ambulation, time to first flatus, time to first oral intake, and chest tube duration were all significantly earlier or shorter in the ERAS group than in the control group (all *P* < .001). Furthermore, the total postoperative hospitalization cost was significantly lower in the ERAS group compared to the control group (¥58,000 vs ¥65,000, *P* = .021).

**Table 3:** Comparison of primary and secondary recovery indicators between the 2 groups during the perioperative period.

Indicator	ERAS group (n = 52)	Control group (n = 52)	Statistical value	*P*-value
Primary indicators				
Postoperative hospital stay (d)	5.0 (4.0, 6.0)	8.0 (7.0, 10.0)	*Z* = −6.123	<.001
Total hospital stay (d)	9.0 (8.0, 11.0)	13.0 (11.0, 15.0)	*Z* = −5.876	<.001
Recovery efficiency indicators				
Time to first ambulation (h)	6.0 (5.0, 8.0)	22.0 (18.0, 26.0)	*Z* = −7.891	<.001
Time to first flatus (h)	28.5 (24.0, 36.0)	42.0 (36.0, 50.0)	*Z* = −5.432	<.001
Time to first oral intake (h)	8.0 (6.0, 10.0)	28.0 (24.0, 32.0)	*Z* = −7.245	<.001
Chest tube duration (d)	3.0 (2.0, 4.0)	5.0 (4.0, 6.0)	*Z* = −5.123	<.001
Total postoperative hospitalization cost (10k RMB)	5.8 ± 1.2	6.5 ± 1.5	*t* = −2.345	.021

ERAS = enhanced recovery after surgery, NRS = Numeric Rating Scale, PONV = postoperative nausea and vomiting.

### 3.3. Comparison of postoperative complications and safety

In terms of safety, the ERAS group demonstrated a lower risk of complications (Table 4). The overall complication rate was significantly lower in the ERAS group compared to the control group (15.4% vs 36.5%, RR = 0.42, 95% CI: 0.21–0.85, *P* = .012). Further analysis indicated that this advantage was primarily reflected in pulmonary complications (9.6% vs 25.0%, RR = 0.38, 95% CI: 0.15–0.99, *P* = .035). There were no statistically significant differences between the 2 groups regarding cardiovascular complications, reoperation rate, or 30-day readmission rate (all *P* > .05).

**Table 4:** Comparison of postoperative complications and safety indicators between the 2 groups, n (%).

Safety indicator	ERAS group (n = 52)	Control group (n = 52)	Relative risk (RR, 95% CI)	*P*-value
Overall complications	8 (15.4%)	19 (36.5%)	0.42 (0.21, 0.85)	.012
Pulmonary complications	5 (9.6%)	13 (25.0%)	0.38 (0.15–0.99)	.035
Pneumonia	3 (5.8%)	7 (13.5%)	–	.189
Atelectasis	2 (3.8%)	5 (9.6%)	–	.242
Prolonged air leak (>5 d)	2 (3.8%)	4 (7.7%)	–	.4
Cardiovascular complications	2 (3.8%)	6 (11.5%)	0.33 (0.07–1.56)	.152
Arrhythmia	2 (3.8%)	5 (9.6%)	–	.242
Other complications	3 (5.8%)	5 (9.6%)	0.60 (0.15–2.37)	.464
Surgical site infection	1 (1.9%)	2 (3.8%)	–	.5
Deep vein thrombosis/pulmonary embolism	0 (0.0%)	1 (1.9%)	–	.315
Anastomotic leak	2 (3.8%)	2 (3.8%)	–	1
Reoperation rate	1 (1.9%)	2 (3.8%)	0.50 (0.05–5.37)	.5
30-d readmission rate	2 (3.8%)	4 (7.7%)	0.50 (0.10–2.58)	.4

ERAS = enhanced recovery after surgery, RR = relative risk.

### 3.4. Postoperative symptom control and patient experience

The ERAS pathway demonstrated significant effects in improving patient experience (Table 5). For postoperative pain, repeated measures analysis of variance revealed a significant time-by-group interaction effect (*P* = .004). At each specific time point, pain scores at rest and during activity were significantly lower in the ERAS group compared to the control group (all *P* < .001). Concurrently, the incidence of postoperative nausea and vomiting (PONV) was significantly lower in the ERAS group (11.5% vs 30.8%, *P* = .020), and patient satisfaction scores were significantly higher (8.9 points vs 7.5 points, *P* < .001).

**Table 5:** Comparison of postoperative symptom control and experience indicators between the 2 groups.

Indicator	ERAS group (n = 52)	Control group (n = 52)	Statistical value	*P*-value
Postoperative pain score (NRS, points)			*F* time × group = 5.678	.004
24 h postop (at rest)	3.5 ± 1.1	4.8 ± 1.3	*t* = −5.432	<.001
48 h postop (at rest)	2.5 ± 0.9	3.6 ± 1.2	*t* = −4.987	<.001
72 h postop (at rest)	1.8 ± 0.7	2.7 ± 1.0	*t* = −4.567	<.001
24 h postop (during activity)	5.2 ± 1.3	6.5 ± 1.4	*t* = −4.789	<.001
Postoperative nausea and vomiting (PONV)	6 (11.5%)	16 (30.8%)	χ^2^ = 5.385	.02
Patient satisfaction (0–10 points)	8.9 ± 1.0	7.5 ± 1.4	*t* = 5.123	<.001

ERAS = enhanced recovery after surgery, NRS = Numeric Rating Scale PONV = postoperative nausea and vomiting.

### 3.5. Long-term prognosis and quality of life

All patients completed the 90-day follow-up with no mortality reported. Quality of life analysis (Table 6) showed significant time-by-group interaction effects for both the global health status/QoL and physical functioning scales of the EORTC QLQ-C30 questionnaire (*P* = .002; *P* = .008), indicating a superior recovery trajectory in the ERAS group. Intergroup comparisons revealed that the ERAS group had significantly higher scores for both global health status and physical functioning at discharge and 30 days postoperatively compared to the control group (all *P* < .01). Regarding symptom control, the ERAS group demonstrated a more favorable improvement trend in cough symptoms (interaction effect *P* = .007), with significantly lower scores than the control group at 30 days postoperatively (*P* = .003). By 90 days postoperatively, the ERAS group also exhibited significantly less dyspnea compared to the control group (*P* = .028).

**Table 6:** Comparison of 90-day postoperative survival and quality of life scores between the 2 groups.

Indicator	ERAS group (n = 52)	Control group (n = 52)	Statistical value/effect size (95% CI)	*P*-value
90-d postoperative survival rate, n (%)	52 (100%)	52 (100%)	–	–
EORTC QLQ-C30 core scale (points)			Group main effect *F* = 8.12	<.001
Global health status/QoL			Time × group interaction *F* = 6.45	.002
At discharge	58.5 ± 12.3	50.2 ± 14.1	MD = 8.3 (3.5–13.1)	.001
30 d postop	68.9 ± 10.5	60.1 ± 12.8	MD = 8.8 (4.3–13.3)	<.001
90 d postop	75.4 ± 8.7	72.8 ± 9.5	MD = 2.6 (−0.8 to 6.0)	.13
Physical functioning			Time × group interaction *F* = 4.98	.008
At discharge	65.8 ± 11.6	58.4 ± 13.2	MD = 7.4 (2.7–12.1)	.002
30 d postop	76.5 ± 9.8	69.3 ± 11.5	MD = 7.2 (3.1–11.3)	.001
90 d postop	82.1 ± 7.2	80.5 ± 8.1	MD = 1.6 (−1.3 to 4.5)	.273
EORTC QLQ-LC13 lung cancer symptom scale (points)				
Cough			Time × group interaction *F* = 5.12	.007
At discharge	38.2 ± 9.5	41.5 ± 10.8	MD = −3.3 (−7.1 to 0.5)	.089
30 d postop	28.6 ± 8.1	33.9 ± 9.4	MD = −5.3 (−8.7 to −1.9)	.003
90 d postop	20.3 ± 6.8	22.1 ± 7.5	MD = −1.8 (−4.5 to 0.9)	.187
Dyspnea			Time × group interaction *F* = 4.05	.019
90 d postop	18.5 ± 6.2	21.3 ± 7.0	MD = −2.8 (–5.3 to −0.3)	.028

CI = confidence interval, EORTC QLQ-C30 = European Organisation for Research and Treatment of Cancer Quality of Life Questionnaire Core 30; EORTC QLQ-LC13 = European Organisation for Research and Treatment of Cancer Quality of Life Questionnaire Lung Cancer 13, ERAS = enhanced recovery after surgery, MD = mean difference, QoL = quality of life.

### 3.6. Subgroup and sensitivity analyses

**Figure 3.**
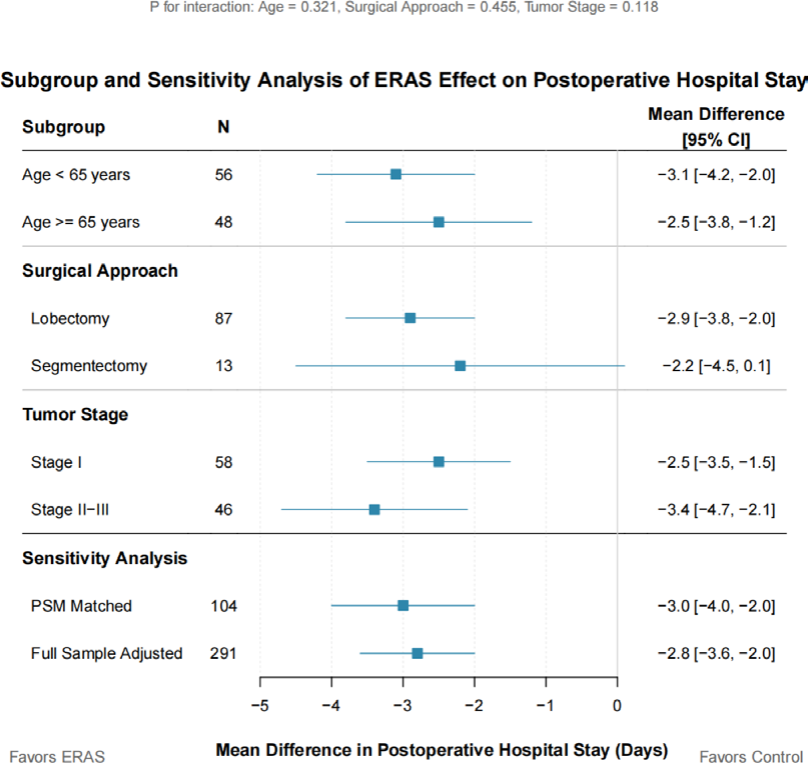
Subgroup and sensitivity analyses of ERAS effect on postoperative hospital stay. Forest plot shows mean differences (days) with 95% CIs. Enhanced recovery after surgery consistently reduced hospital stay across all subgroups (blue), with nonsignificant interaction effects. Sensitivity analyses (burgundy) confirmed result robustness. Squares represent effect estimates; horizontal lines show 95% CIs. CI = confidence intervals, ERAS = enhanced recovery after surgery, PSM = propensity score matching.

Subgroup analyses showed (Table 7 and Figure 3) that the benefit of ERAS in reducing postoperative hospital stay was consistent across patients of different ages, surgical methods, and tumor stages, with no statistically significant interactions observed between subgroups (all *P* > .05). Sensitivity analyses further confirmed the robustness of the results: using the full pre-matched sample for multivariate linear regression analysis, after adjusting for confounding factors, ERAS remained an independent factor associated with shortened postoperative hospital stay. The effect size was highly consistent with the primary analysis results (mean difference: −2.8 days [95% CI: −3.6 to −2.0] vs −3.0 days).

**Table 7:** Subgroup and sensitivity analyses of postoperative hospital stay.

Analysis type	Subgroup/model	Patient number (n)	Effect size (95% CI)	*P*-value for interaction
Subgroup analysis				
	Age			.321
	<65 yr	56	−3.1 (−4.2 to −2.0)	
	≥65 yr	48	−2.5 (−3.8 to −1.2)	
	Surgical approach			.455
	Lobectomy	87	−2.9 (−3.8 to −2.0)	
	Segmentectomy	13	−2.2 (−4.5 to 0.1)	
	Tumor stage			.118
	Stage I	58	−2.5 (−3.5 to −1.5)	
	Stage II–III	46	−3.4 (−4.7 to −2.1)	
Sensitivity analysis				
	PSM matched sample (primary analysis)	104	−3.0 (−4.0 to −2.0)	<.001
	Multivariate linear regression model (full pre-match sample)	291	−2.8 (−3.6 to −2.0)	<.001

CI = confidence interval, PSM = propensity score matching.

## 4. Discussion

This study systematically evaluated the effect of the enhanced recovery after surgery (ERAS) pathway in patients undergoing radical lung cancer surgery by employing PSM to effectively balance baseline characteristics. The results consistently demonstrated that the ERAS pathway significantly improved perioperative outcomes. This finding aligns with previous research in fields such as colorectal and orthopedic surgery,^[11]^ further confirming the applicability of the ERAS concept in thoracic surgery. Specifically, patients in the ERAS group had significantly shorter postoperative and total hospital stays. This benefit is likely attributable to the synergistic effects of multimodal interventions: preoperative carbohydrate loading reduced insulin resistance,^[12]^ intraoperative precise fluid management maintained physiological homeostasis, and postoperative early ambulation and oral intake effectively promoted the recovery of gastrointestinal and overall physiological function.^[13]^ These measures collectively broke the vicious cycle of recovery delay caused by prolonged fasting, bed rest, and other factors inherent in traditional management models.

Regarding safety, the ERAS pathway demonstrated significant advantages, particularly by reducing the risk of pulmonary complications by 62%. This benefit may stem from multiple mechanisms: systematic preoperative respiratory function exercises enhanced patients’ pulmonary reserve,^[14]^ while standardized postoperative breathing exercises and early mobilization effectively prevented atelectasis and pulmonary infections.^[14]^ Concurrently, optimized pain management strategies, especially the application of regional analgesia techniques, not only reduced opioid consumption and their depressive effect on the respiratory center but also enabled patients to perform effective coughing, expectoration, and early mobilization sooner and more effectively, which is crucial for preventing pulmonary complications.^[13,15]^ Furthermore, the significantly lower incidence of PONV in the ERAS group may be related to measures such as intraoperative prophylactic use of antiemetics, avoidance of excessive opioids, and early oral intake.^[15]^ These interventions collectively improved the overall recovery experience for patients.

Beyond statistical significance, the reduction in postoperative and total hospital stay observed in the ERAS group carries substantial clinical and health-economic implications. Shortened hospitalization directly improves hospital bed turnover and resource utilization, which is particularly important in high-volume thoracic surgery centers where medical resources are often limited. From the patient perspective, a shorter length of stay reduces direct medical expenses related to hospitalization as well as indirect economic burdens, such as loss of productivity and caregiver costs. Moreover, earlier discharge may facilitate timelier initiation of adjuvant therapies, including chemotherapy or targeted treatment, which is crucial for optimizing oncological treatment continuity in lung cancer patients. Collectively, these advantages indicate that the ERAS nursing pathway not only accelerates postoperative recovery but also enhances the overall efficiency of perioperative management, providing meaningful value at both the individual patient level and the healthcare system level.^[16]^

Through subgroup analysis, we found that the benefits of the ERAS pathway remained consistent across patients of different ages, surgical approaches, and tumor stages, enhancing the generalizability of the study findings. Simultaneously, the results of the sensitivity analysis further validated the robustness of the primary conclusions, indicating that the clinical value of ERAS remains stable even under different statistical models and analytical methods.^[17]^ These methodologically rigorous designs provide more persuasive evidence for the application of ERAS in lung cancer surgery compared to previous studies.

Several limitations of this study should be acknowledged. First, although PSM was employed to balance measured baseline characteristics and reduce selection bias, residual confounding from unmeasured variables cannot be completely excluded. Factors such as individual surgeon experience, variations in perioperative anesthesia management, and baseline pulmonary function parameters were not fully captured in the retrospective dataset and may have influenced postoperative recovery and complication rates. Second, this was a single-center retrospective study, and institutional practices, patient characteristics, and ERAS implementation protocols may differ across centers. Therefore, the external validity and generalizability of our findings may be limited. Future multicenter, prospective randomized controlled studies with more comprehensive perioperative variables are warranted to further validate the observed benefits of the ERAS nursing pathway.

In summary, this study, through its rigorous methodological design, confirms that for lung cancer patients undergoing radical surgery, implementing the ERAS nursing pathway can safely and effectively accelerate postoperative recovery, significantly shorten hospital stay, reduce the risk of complications, and simultaneously improve patients’ symptom experience and QoL. These findings provide strong evidence-based support for the promotion and application of the ERAS pathway in the field of thoracic surgery. It is recommended that this optimized management model be systematically implemented in clinical practice, tailored to individual patient characteristics.

## Author contributions

**Conceptualization:** Yanmei Chen, Dequn Chen, Yayuan Wang, Yuanqiang Zhang.

**Data curation:** Yanmei Chen, Dequn Chen, Yayuan Wang, Yuanqiang Zhang.

**Formal analysis:** Yanmei Chen, Dequn Chen, Yayuan Wang, Yuanqiang Zhang.

**Funding acquisition:** Yanmei Chen, Yuanqiang Zhang.

**Investigation:** Yanmei Chen, Yuanqiang Zhang.

**Writing – original draft:** Yuanqiang Zhang.

**Writing – review & editing:** Yuanqiang Zhang.
